# Facile Fabrication of Superhydrophobic Cross-Linked Nanocellulose Aerogels for Oil–Water Separation

**DOI:** 10.3390/polym13040625

**Published:** 2021-02-19

**Authors:** Qianqian Shang, Jianqiang Chen, Yun Hu, Xiaohui Yang, Lihong Hu, Chengguo Liu, Xiaoli Ren, Yonghong Zhou

**Affiliations:** 1 Institute of Chemical Industry of Forest Products, CAF, Key Lab. of Biomass Energy and Material, Key and Open Lab. of Forest Chemical Engineering, SFA, National Engineering Lab. for Biomass Chemical Utilization, Nanjing 210042, China; shangqianqian@icifp.cn (Q.S.); huyun@icifp.cn (Y.H.); yxh@icifp.cn (X.Y.); hlh@icifp.cn (L.H.); renxiaoli@icifp.cn (X.R.); 2 Co-Innovation Center of Efficient Processing and Utilization of Forest Resources, Nanjing Forestry University, Nanjing 210037, China; chenjq@njfu.edu.cn; 3 College of Biology and the Environment, Nanjing Forestry University, Nanjing 210037, China

**Keywords:** nanocellulose, aerogels, cross-linking, superhydrophobic, oil adsorption

## Abstract

A facile and environmental-friendly approach was developed for the preparation of the cross-linked nanocellulose aerogel through the freeze-drying process and subsequent esterification. The as-prepared aerogel had a three-dimensional cellular microstructure with ultra-low density of 6.05 mg·cm^−3^ and high porosity (99.61%). After modifying by chemical vapor deposition (CVD) with hexadecyltrimethoxysilane (HTMS), the nanocellulose aerogel displayed stable super-hydrophobicity and super-oleophilicity with water contact angle of 151°, and had excellent adsorption performance for various oil and organic solvents with the adsorption capacity of 77~226 g/g. Even after 30 cycles, the adsorption capacity of the nanocellulose aerogel for chloroform was as high as 170 g/g, indicating its outstanding reusability. Therefore, the superhydrophobic cross-linked nanocellulose aerogel is a promising oil adsorbent for wastewater treatment.

## 1. Introduction

Oil spill and wastewater have posed severe threats to the ecological environment and human health [1,2,3]. Traditional response tactics for oil spill cleanup include in-situ combustion, gas flotation, centrifugation, chemical precipitation, and so on [4,5,6]. However, most of these response countermeasures suffer from inefficient separation, lower selectivity, and energy-extensive consumption, which limit their wide application in oil/water separation. 

Recently, aerogels with remarkable comprehensive performances have aroused considerable attention in catalyst supports, sensors, thermal super-insulators and biomedical applications [7,8,9]. In particular, their low density, lightweight, high specific surface area, and porosity make them very suitable to remove hazardous pollutants and oils from water [10,11,12]. To improve the adsorption selectivity, it is essential to control the wetting properties of aerogels. Inspired by lotus leaf, the superhydrophobic surfaces have received great attention in oil spill treatment [13,14,15]. Many reports have proved that appropriate rough morphology and low surface energy are indispensable to fabricate superhydrophobic materials [16,17,18]. A bottom-up assembly process is commonly used for the preparation of superhydrophobic aerogels [19,20]. So far, a variety of superhydrophobic aerogels have been developed, including silica-based aerogels [21,22], carbon-based aerogels [23,24,25,26], cellulose-based aerogels [27,28,29,30,31,32], and boron nitride aerogels [33], etc. Among them, the cellulose aerogel has been considered as the most promising aerogel.

As a renewable bio-resource, cellulose has been widely utilized in biomedicine, cosmetics, energy storage, and water purification [34,35,36,37,38,39,40,41,42,43,44]. Cellulose nanofibril (CNF) extracted from bulk cellulose is a string-like nanomaterial with some unique characteristics, including renewability, biocompatibility, biodegradability, high elastic modulus, and excellent thermal stability [45,46,47,48,49,50]. Over the past decades, increasing attention has been paid to the exploitation of CNF to fabricate cellulose-based aerogels for oil/water separation [51,52,53,54]. However, CNF-based aerogels can be easily disintegrated by exposing them to liquid water due to the weak hydrogen bond interaction between nanocellulose, which limits their large-scale practical applications.

To overcome the above shortcomings, several covalent cross-linking methods are developed to improve the aerogels’ structural stability. Addition of cross-linking agents including silane [55], maleic acid [56], and polyamide-epichlorohydrin resin [57] to the nanocellulose before fabricating drying materials has been considered as the common method. 1.2.3.4-Butanetetracarboxylic acid (BTCA) is a kind of organic polycarboxylic acid, which can form covalent ester bonds with cellulose. Though organic polycarboxylic acid has been used for cross-linking modification of nanocellulose aerogels [58], the fabrication of superhydrophobic cross-linked cellulose aerogel for oil spill treatment is still needed.

Here, a superhydrophobic cross-linked nanocellulose aerogel has been created by means of a freeze-drying process and post-cross-linking technology using BTCA as a cross-linking agent, followed by the hydrophobic modification of hexadecyltrimethoxysilane (HTMS). The as-prepared superhydrophobic nanocellulose aerogel had the ultra-light weight (7.1 mg·cm^−3^), the high porosity (99.55%) and the super-hydrophobicity/super-oleophilicity (water contact angle as high as 151°). The superhydrophobic aerogel demonstrated to be an ideal adsorbent for removing oils from water with high selectivity and excellent recyclability (at least 30 cycles). The adsorption capacities toward variety of oil and organic solvents could reach up to 77~226 g/g, which are higher than most oil adsorbents reported in recent years, indicating its great potential in wastewater treatment.

## 2. Materials and Methods

### 2.1. Materials

CNF was purchased from Zhongshan NanoFC Bio-materials Co., Ltd. (Zhongshan, China). Sodium hypophosphite monohydrate (SHP, 98%), BTCA (99%) and HTMS (85%) were obtained from Aladdin (Shanghai, China). Toluene, silicone oil, n-hexane, cyclohexane, 1,2-dichloromethane, chloroform, dimethyl sulfoxide (DMSO), dimethyl formamide (DMF), acetone, and ethanol were obtained from Sinopharm Chemical Reagent Co., Ltd. (Shanghai, China) Olive oil and gasoline were purchased from a local store(Nanjing, China). All the chemicals were used as received. Milli-Q water was used in all experiments.

### 2.2. Fabrication of Cross-linked Cellulose Aerogels

BTCA (0.05, 0.1, and 0.15 g) was added into the CNF aqueous suspensions (100 mL, 0.5% g/mL) with the mass ratio of CNF to BTCA as 10/1, 10/2, and 10/3. Then, the mixtures were stirred vigorously for 6 h to obtain the homogenous suspensions. Subsequently, SHP (the mass ratio of SHP to BTCA is 1:1) was added into the mixture with further stirring for 30 min. The as-prepared solution was transferred into several molds and put into a lyophilizer (Scientz-10ND, Ningbo Scientz Biotechnology Co. Ltd., Ningbo, China) at –50 °C for freeze-drying for 72 h. Thereafter, the obtained aerogels were treated at 170 °C for 5 min to form sufficient covalent reaction. These aerogels were named as CNF/BTCA_10/1_, CNF/BTCA_10/2_, and CNF/BTCA_10/3_. For comparison, pure CNF aerogel was fabricated without BTCA and SHP.

### 2.3. Preparation of Superhydrophobic Cellulose Aerogel

Superhydrophobic cellulose aerogel was prepared via a CVD method. Typically, HTMS (1 mL) was added into a stainless-steel reactor with a tetrafluoroethylene lining. A portion of CNF/BTCA aerogel (0.10 g) was placed on a mesh interlayer, which was decorated in the container to prevent the aerogel from contacting the bottom HTMS directly. After being sealed, the container was placed in an oven and heated for 3 h at 120 °C to obtain the superhyrophobic cellulose aerogel (Si-CNF/BTCA)aerogel.

### 2.4. Characterizations

Fourier Transform infrared spectroscopy (FT-IR) analysis was performed on a Nicolet iS 10 spectrophotometer (Thermofisher Scientific, Waltham, MA, USA) with the scan range of 4000−400 cm^−1^. The surface morphologies and surface elements of the aerogels were characterized by using an S-3400N scanning electron microscopy (SEM, Hitachi, Tokyo, Japan) equipped energy dispersive X-ray spectroscopy (EDX) operated at 5.0 kV without sputtered Au. Thermogravimetric analyses (TGA, Netzsch, Bavaria, Germany) of the aerogels were carried out on a STA409PC thermogravimetry analyzer. Chemical compositions were evaluated by using an ESCALAB250Xi X-ray photoelectron spectroscopy (XPS, Thermofisher Scientific, Waltham, MA, USA). Water contact angle (WCA) was recorded on a contact angle meter (CV-705B, CVOK, Dongguan, China) using 2 μL of water. 

### 2.5. Density and Porosity 

The density of the aerogel was calculated according to Equation (1):

(1) $$\rho_{a=}\frac{m}{V}$$

where *m* represents the aerogel weight and *V* represents the aerogel volume.

The porosity (*P*) of the aerogel was evaluated using Equation (2):

(2) $$P\;(\%)\;=\;\frac{\rho_{s}-\rho_{a}}{\rho_{s}}\times \;100\%\;$$

where *ρ*_a_ and *ρ*_s_ represent the aerogel and cellulose densities, respectively.

### 2.6. Oil Adsorption Capacity

The oil adsorption performance of the superhydrophobic Si-CNF/BTCA aerogel was measured by using various oils and organic solvents. Si-CNF/BTCA aerogel was dried in an oven and weighted before the oil adsorption test. Afterwards, the Si-CNF/BTCA aerogel was immersed in oil to reach saturation adsorption, and then taken out and weighed. At least three tests were carried out for each sample. The oil adsorption capacity, *C*, of the Si-CNF/BTCA aerogel was evaluated using Equation (3):

(3) $$C\;\left(g/g\right)\;=\frac{m-m0}{m0}$$

where *m*_0_ represents the initial aerogel weight and m represents the aerogel weight after absorption.

## 3. Results and Discussion

### 3.1. Fabrication of Superhydrophobic Cross−Linked Cellulose Aerogels

**Scheme 1:**
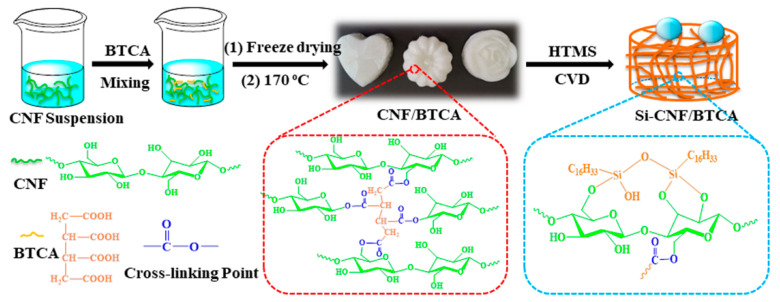
Schematic illustration for the superhyrophobic cellulose aerogel (Si-CNF/BTCA) aerogel.

The fabrication process of the superhydrophobic cross-linked CNF (Si-CNF/BTCA) aerogel with turntable shape is schematically illustrated in Scheme 1. The cross-linking agent BTCA was firstly added into the CNF aqueous solution to form the BTCA-CNF composite solution. As presented in Scheme 1, CNF molecular chains are rich in hydroxyl groups, while the BTCA molecule has four carboxyl groups. After the freeze-drying process, the composite solution was converted into a CNF/BTCA aerogel. Various shapes of the as-prepared aerogels could be manipulated using molding. Thereafter, the ester linkages were generated between the carboxyl groups of BTCA and the hydroxyl groups of cellulose in the high temperature. With the further hydrophobic modification of HTMS via the CVD process, the silanol of HTMS could react with the residual hydroxyl groups of cellulose [59], and the superhydrophobic Si-CNF/BTCA aerogel was obtained.

### 3.2. The Apparent Morphologies and Water-Durable Properties

**Figure 1:**
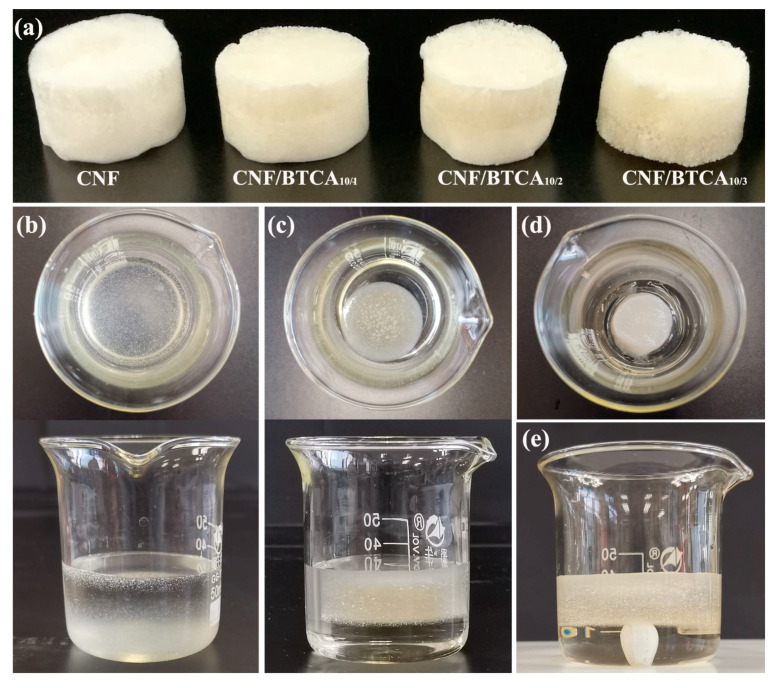
(**a**) Morphologies of the pure CNF, CNF/BTCA_10/1_, CNF/BTCA_10/2_, and CNF/BTCA_10/3_ aerogels. (**b**) Photographs of the pure CNF aerogel disintegrated in water. (**c**) Photographs of the cross-linked CNF/BTCA_10/1_ aerogel keeping intact in water. (**d**) The cross-linked CNF/BTCA_10/1_ aerogel remaining intact after pouring out the excess water and (**e**) in stirring water.

The effect of the BTCA dosage on the aerogel morphologies was investigated as shown in Figure 1a. When the mass ratio of CNF to BTCA was 10/1 (CNF/BTCA_10/1_), the morphology of aerogels was similar to that of the pure CNF aerogel. However, with the increase of BTCA dosage, the color of the aerogels became yellow, and the structure of the aerogels became loose, which was due to the increasing cross-linking degree resulting in the decrease of the flexibility of aerogels. In addition, with the increase of the BTCA dosage, the densities of these aerogels increased while the porosities tended to decrease (Supplementary Figure S1). Therefore, CNF/BTCA_10/1_ aerogel with the good flexibility and high porosity was chosen for further investigation.

The pure CNF aerogel and CNF/BTCA_10/1_ aerogel were each impregnated in ultrapure water to investigate their water-durable properties, as shown in Figure 1b,c. The pure CNF aerogels without cross-linking were easy to disintegrate after swaying in water overnight (Figure 1b), because the hydrogen-bond interaction between CNF was easy to destroy. However, the cross-linked CNF/BTCA_10/1_ aerogel could maintain a complete cylinder-shape in water after one day (Figure 1c) and even after magnetic stirring for 1 h (Figure 1e). Furthermore, as shown in Figure 1d, after pouring out the excess water, the CNF/BTCA_10/1_ aerogel still retained its three-dimensional porous structure, indicating the enhanced structural stability of CNF/BTCA_10/1_ aerogel by chemical cross-linking reaction.

### 3.3. The Aerogel Wettability 

**Figure 2:**
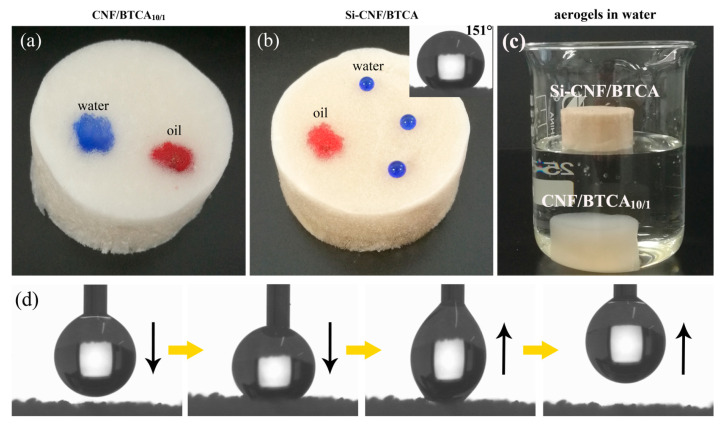
The wettability of oil and water on the CNF/BTCA_10/1_ aerogel (**a**) and Si-CNF/BTCA aerogel (**b**). (**c**) Photograph of CNF/BTCA_10/1_ and Si-CNF/BTCA aerogels in water. (**d**) Photographs of the contact process between the water droplet and Si-CNF/BTCA aerogel.

The contact angles of the oil and water droplets on the CNF/BTCA_10/1_ and Si-CNF/BTCA aerogels were revealed in Figure 2. The oil and water droplets could be completely absorbed by the CNF/BTCA_10/1_ aerogel (Figure 2a), which indicates the superhydrophilicity and super-oleophilicity of the CNF/BTCA_10/1_ aerogel. For Si-CNF/BTCA aerogel, the oil droplet was adsorbed immediately upon touching the aerogel, while the water droplet was repelled to form a ball-like shape on the aerogel surface (Figure 2b). Using the modification of 1 mL HTMS, the WCA of the Si-CNF/BTCA aerogel was 151°, which confirmed its super-hydrophobicity. Increasing the amount of HTMS, the WCA did not increase significantly (151.2°), which might be due to the saturation of the reaction between HTMS and CNF. Figure 2c shows that the Si-CNF/BTCA could float on the water surface, while the CNF/BTCA_10/1_ could sink into water. These results proved the successful preparation of the superhydrophobic cross-linked Si-CNF/BTCA. To study the water proofing property of the Si-CNF/BTCA, the contact process of the water droplet and the aerogel was recorded, as shown in Figure 2d. Even after the squeezing process, the water droplet could completely leave the surface of the aerogel without any residue, indicating the low adhesion to water. 

### 3.4. Chemical Structure and Thermostability

**Figure 3:**
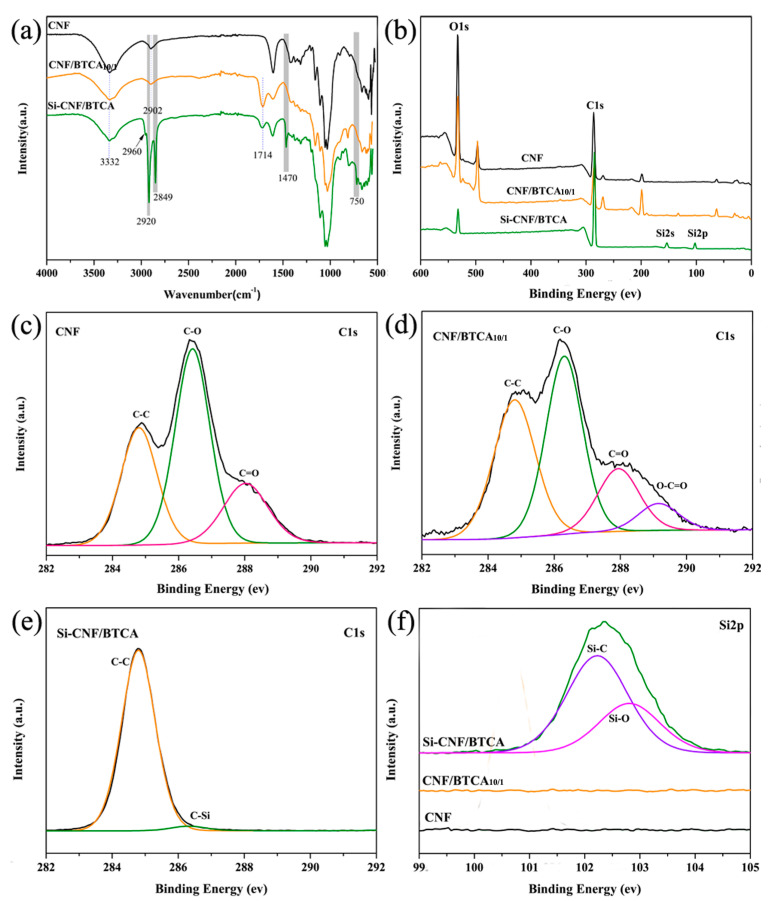
Fourier Transform infrared spectroscopy (FT-IR) (**a**) and X-ray photoelectron spectroscopy (XPS) (**b**) spectra for the aerogels. C1s spectra for the pure CNF (**c**), CNF/BTCA_10/1_ (**d**), and Si-CNF/BTCA (**e**) aerogels. (**f**) Si2p spectra for the aerogels.

The chemical structure of the samples was analyzed using FT-IR and XPS. Figure 3a reveals the FT-IR spectra of the aerogels. For the pure CNF and CNF/BTCA_10/1_ aerogels, the FT-IR spectra of both aerogels display a broad band peak centered at 3332 cm^−1^ for the O-H group, and a characteristic band at 2902 cm^−1^ for the stretching vibration of the C-H group. When BTCA was added, the esterification reaction between CNF and polycarboxylic acids occurred [58]. The hydroxyl groups of CNF reacted with one or two carboxyl groups in BTCA to form ester bonds for chemical cross-linking. For the CNF/BTCA_10/1_ aerogel, a new characteristic absorption peak for the ester carbonyl group (C=O) was detected at 1714 cm^−1^ and the stretching vibration peak for O-H group of CNF became weaker. This indicates that the ester bonds between CNF and BTCA were successfully generated. It can also be speculated that the internal network structure of the CNF/BTCA_10/1_ aerogel was formed by hydrogen bonding and chemical cross-linking between BTCA and CNF.

With the hydrophobic modification of HTMS, new peaks for methyl at 2960 cm^−1^, and for methylene at 2920 cm^−1^ and 2849 cm^−1^ corresponding to the long carbon chain of HTMS, were observed in the Si-CNF/BTCA aerogel. At the same time, the peak for C-H bending vibration was also detected at 1470 cm^−1^, which resulted from the modification of HTMS on the cellulose surface. In addition, the peak at 750 cm^−1^ corresponded to the asymmetric stretching vibration of Si-C and -CH_3_ in siloxane compounds. The typical peak of Si-O-Si at 1000–1130 cm^−1^ overlapped with that of cellulose C-O bond [60]. These results demonstrated the formation of the covalent bond between CNF and HTMS, which was beneficial to maintain the hydrophobic stability of the aerogel in practical application.

XPS was employed to analyze the surface composition of the aerogels, as revealed in Figure 3b. It can be seen that C and O elements are the main compositions for the pure CNF and CNF/BTCA_10/1_ aerogels. A small amount of Cl (198.74 eV) and P (132.91 eV) elements were also detected in the pure CNF and CNF/BTCA_10/1_ aerogels, which were caused by the use of sodium hypochlorite and sodium hypophosphite in the preparation process of CNF and the cross-linked aerogel. For the Si-CNF/BTCA aerogels, the peak of Si element was detected, indicating the presence of the polysiloxane on the surface of the superhydrophobic aerogel. The C1s spectra of the aerogels were shown in Figure 3c–e. The C1s peak in the pure CNF aerogel was divided into three binding forms at 284.83 eV for C-C bond, at 286.43 eV for C-O bond, and at 288.03 eV for C=O bond (Figure 3c). Compared with the pure CNF aerogel, a new peak at 289.63 eV for the O-C=O group was detected in the CNF/BTCA_10/1_ aerogel (Figure 3d). The presence of the ester bond further confirmed the occurrence of the cross-linking reaction between CNF and BTCA. For Si-CNF/BTCA aerogel, the C1s peak was only divided into the two bindings at 284.80 eV for C-C bond and at 286.32 eV for C-Si bond (Figure 3e), which were derived from the HTMS. It is known that the XPS can be utilized to measure the elemental component of the material surface in the range of 1–10 nm. Based on the change of the C1s spectra of these aerogels, we can deduce that a dense polysiloxane layer was formed on the aerogel surface after the hydrophobic modification. The Si2p spectrum detected in the Si-CNF/BTCA aerogel further verified this conclusion. 

**Figure 4:**
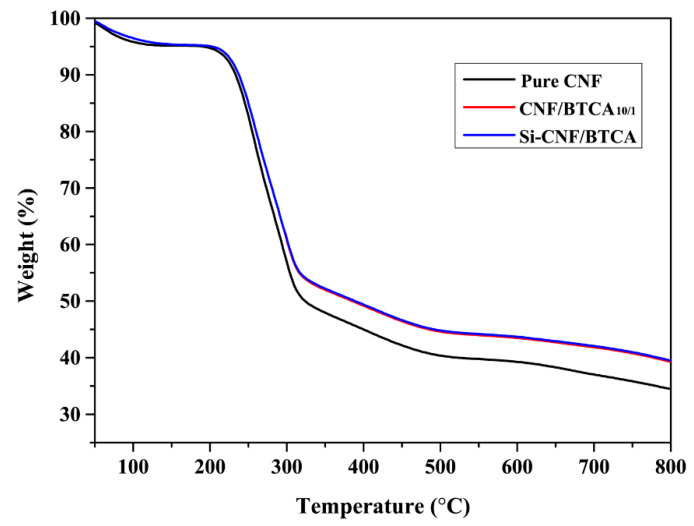
Thermogravimetric analyses (TGA) curves of the pure CNF, CNF/BTCA_10/1_, and Si-CNF/BTCA aerogels.

The thermal stability of the aerogels was also characterized, as shown in Figure 4. It was found that the CNF/BTCA_10/1_ and Si-CNF/BTCA aerogels displayed higher decomposition temperatures owing to the presence of the cross-linking bonds. The residual rates of these aerogels at 800 °C were 34.5%, 39.3%, and 39.5% for the pure CNF, CNF/BTCA_10/1_, and Si-CNF/BTCA aerogels, respectively. The increased residues for the CNF/BTCA_10/1_ and Si-CNF/BTCA aerogels might be caused by the addition of the cross-linking agent and catalyst, and the modification of HTMS.

### 3.5. The Morphology and Porosity

**Figure 5:**
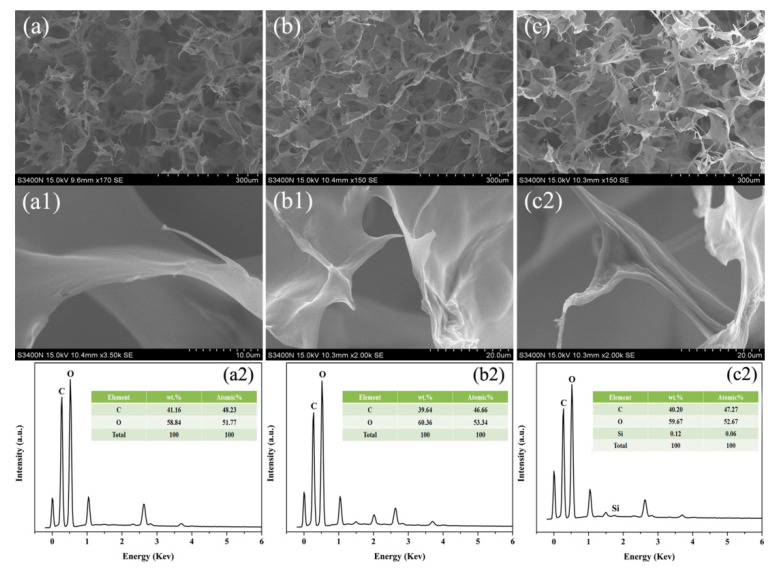
Scanning electron microscopy (SEM) images of the pure CNF (**a**,**a1**), CNF/BTCA_10/1_ (**b**,**b1**), and Si-CNF/BTCA (**c**,**c1**) aerogels. Energy dispersive X-ray spectroscopy(EDX) spectra of the pure CNF (**a2**), CNF/BTCA_10/1_ (**b2**), and Si-CNF/BTCA (**c2**) aerogels.

Figure 5 reveals the SEM images and EDX spectra of the aerogels. It can be found that all the samples displayed the porous three-dimensional honeycomb-like structure (Figure 5a–c). The freezing process of the CNF-based suspension could lead to the segregation of CNF from the growing ice crystals. After the ice sublimation, large and open pores were formed. The aerogel skeletons were composed of fibers and sheets. The fibers were formed due to the intertwining of the CNF with large aspect ratio and the fragments were formed because of the assembly of the CNF during the freeze-drying process. The skeletons of the pure CNF and CNF/BTCA_10/1_ aerogels were smooth (Figure 5a1 and b1). For the Si-CNF/BTCA aerogel, the wrinkled structure was observed on the pore wall and cellulose skeleton, which might be caused by the formation of hydrophobic layers on the surface of aerogel (Figure 5c1). The formed rough structure was beneficial for achieving the super-hydrophobicity. In the EDX spectra (Figure 5a2, b2, and c2), it is observed that the main elements for the pure CNF and CNF/BTCA_10/1_ aerogels were C and O. For Si-CNF/BTCA aerogel (Figure 5c2), Si element was detected, which further proved the successful modification of HTMS.

Table 1 shows the apparent densities and porosities of the aerogels. The apparent density of the pure CNF aerogel was 5.76 mg cm^−3^, and the porosity was up to 99.63%. The apparent densities of the aerogels increased and the porosities slightly decreased with the addition of BTCA and hydrophobic modification of HTMS. However, the porosity of the Si-CNF/BTCA aerogel was still higher than that of the inorganic aerogels and other organic aerogels [21,61]. The light weight and high porosity of the aerogels were very favorable for their application as oil adsorbent.

### 3.6. Oil Absorption Performance

**Figure 6:**
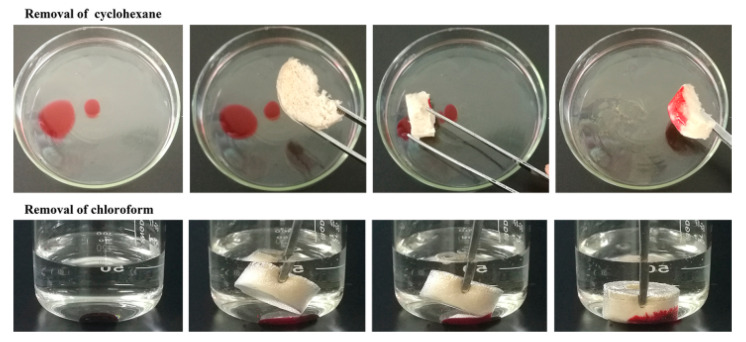
Adsorption of red-colored cyclohexane and chloroform from the surface and bottom of water using the Si-CNF/BTCA.

Due to its super-hydrophilicity/super-oleophilicity, low density, porosity, and robust stability, the Si-CNF/BTCA can be used as the oil adsorbent. Figure 6 shows the selective oil absorption performance of Si-CNF/BTCA aerogel from water surface and underwater. For the cyclohexane/water mixture, the light oil (cyclohexane dyed with red oil) floated on the water. The Si-CNF/BTCA aerogel was then put in the cyclohexane/water mixture. It can be found that the Si-CNF/BTCA aerogel floated on the water and selectively adsorbed cyclohexane without water. Because of the super-oleophilicity of the Si-CNF/BTCA aerogel, the oil with low surface free energy could be adsorbed spontaneously into the internal pores of the aerogel, and the water with high surface free energy was repelled. To investigate the oil–water selectivity of the Si-CNF/BTCA aerogel, chloroform (dyed with red oil) was chosen as the weight oil and mixed with water. When the Si-CNF/BTCA was put in water, the air trapped in the holes of the aerogel formed a mirror-like phenomenon. Once it was exposed to chloroform, chloroform was immediately adsorbed by the Si-CNF/BTCA aerogel and the separation of chloroform and water was realized. Due to its low density and superhydrophobic property, the Si-CNF/BTCA could return to the water surface after adsorbing chloroform, which would be beneficial to the fast recovery of Si-CNF/BTCA aerogel in practical oil–water separation application. 

**Figure 7:**
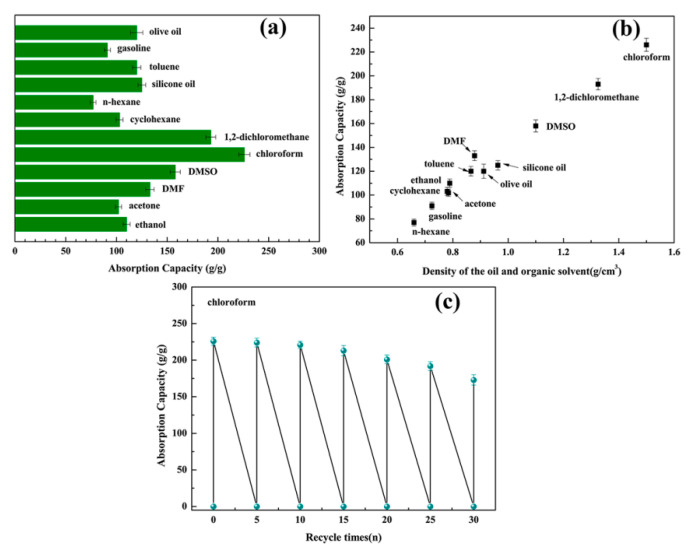
(**a**) Adsorption capacities of Si-CNF/BTCA aerogel for various organic liquids. (**b**) The correlation of the adsorption capacity of the Si-CNF/BTCA and the density of adsorbed oil and organic solvents. (**c**) Recyclability of Si-CNF/BTCA aerogel for the adsorption of chloroform within 30 cycles.

In order to assess the oil adsorption capacity of the Si-CNF/BTCA, various oils and organic solvents, including olive oil, gasoline, toluene, silicone oil, n-hexane, cyclohexane, 1,2-dichloromethane, chloroform, DMSO, DMF, acetone, and ethanol, were studied, as shown in Figure 7. It can be found that the Si-CNF/BTCA had excellent adsorption performance for these organic liquids. The adsorption capacities of Si-CNF/BTCA aerogel were in the range of 77~226 g/g (Figure 7a), which was concerned with the densities of the adsorbed organic liquids. There was an approximate linear relation of the adsorption capacity and the density (Figure 7b), which demonstrated that the oil adsorption performance of Si-CNF/BTCA aerogel was dominated by its porosity.

The reusability of oil absorption material is an important property for practical application. The reusability of the Si-CNF/BTCA was investigated by washing the aerogel with excessive toluene. The aerogel was subsequently vacuum-dried at 60 °C for 10 h and weighed for further use. As displayed in Figure 7c, the adsorption capacity of the Si-CNF/BTCA for chloroform slightly decreased from 226 to 170 g/g after 30 cycles, which indicated the excellent adsorption performance and stable reusability of the Si-CNF/BTCA aerogel. It is notable that the Si-CNF/BTCA aerogel showed higher adsorption capacity than other oil absorption aerogels. The detailed comparison is shown in Table 2. Although HTMS has been used for the fabrication of the hydrophobic nanocellulose aerogel [62], the Si-CNF/BTCA aerogel displayed better adsorption performance and recyclability because of its higher porosity and stable cross-linked structure.

## 4. Conclusions

In summary, the superhydrophobic cross-linked Si-CNF/BTCA aerogel was successfully prepared through sequential freeze-drying, esterification cross-linking, and hydrophobic modification. After cross-linking, the aerogel exhibited enduring water-durability because of the formation of the ester bonds. With the modifying of HTMS, Si-CNF/BTCA aerogel showed super-hydrophobicity/super-oleophilicity and exhibited extraordinary adsorption capacity (77~226 g/g) towards various oil and organic solvents. The excellent adsorption property could be reserved in repetitive use. The sustainable materials, low cost, and excellent adsorption performance made the Si-CNF/BTCA aerogel a promising alternative to commonly used adsorbents in the oily wastewater treatment.

## Figures and Tables

**Table 1:** Details of apparent densities (ρ_a_) and porosities of the aerogels.

Samples	ρ_a_ (mg/cm^3^)	Porosity (%)
CNF	5.42	99.72
CNF/BTCA_10/1_	6.05	99.61
Si-CNF/BTCA	7.10	99.55

**Table 2:** Comparison of the fabrication methods and the absorption performances of the different superhydrophobic aerogels.

Samples	Fabrication Method	Absorption Capacity	Recyclability	Reference
Lignin-based carbon aerogel	Polymerization, anneal	32–34 g/g	―	[23]
Exfoliated-graphene carbon aerogel	Hydrazine hydrate, carbonization	40.2–80.9 g/g	―	[24]
Carbon@SiO_2_@MnO_2_ aerogel	Carbonization, hydrothermal, hydrophobic modification	60–120 g/g	9 cycles	[25]
Nanocrystalline cellulose aerogel	Freeze-drying	Toluene: ~16 g/g	10 cycles	[27]
Superhydrophobic cellulose/poly(vinyl alcohol) composite aerogels	Freeze-drying, chemical vapor deposition (CVD) of methyltrichlorosilane	28–65 g/g	―	[29]
Superlight nanocellulose aerogel foam	Emulsification, freeze-drying	Cyclohexane: 206.69 g/g Ethyl acetate: 194.75 g/g Vacuum pump oil: 145.20 g/g	―	[30]
Copper nanoparticles-coated cellulose aerogel	Depositing Cu nanoparticles, freeze-drying	67.8–164.5 g/g	10 cycles	[31]
Cotton-cellulose aerogel	Freeze-drying, silanization of methyltrimethoxysilane	Machine Oil: 72.3 g/g Dichloromethane: 94.3 g/g	5 cycles	[52]
Superabsorbent from micro-fibrillated cellulose fibers	Ball-milling, freeze-drying, CVD of methyltrimethoxysilane	88–228 g/g	30 cycles	[53]
Hydrophobic nanocellulose-based aerogel	Freeze-drying, CVD of hexadecyltrimethoxysilane	Motor oil: 55.7–78.8 g/g Cooking oil: 94.6–162.4 g/g	20 cycles	[62]
Superhydrophobic cross-linked nano-cellulose aerogels	Freeze-drying, cross-linking, CVD of hexadecyltrimethoxysilane	77–226 g/g	30 cycles	This work

## Data Availability

The data presented in this study are available on request from the corresponding author.

## Supplementary Materials

The following are available online at https://www.mdpi.com/2073-4360/13/4/625/s1, Figure S1: The variation of the density and porosity of the aeroges with the increasing BTCA dosage.
