# Supplementary material for: Facile Fabrication of Superhydrophobic Cross-Linked Nanocellulose Aerogels for Oil–Water Separation
Source: Polymers (Basel). 2021 Feb 19;13(4):625. doi: 10.3390/polym13040625 (PMC7921982; doi:10.3390/polym13040625)
Supplement: Supplementary file 1 [file polymers-13-00625-s001.pdf]

Facile fabrication of superhydrophobic cross-linked nanocellulose aerogels for oil-water separation

Qianqian Shang[1, 2], Jianqiang Chen[2, 3], Yun Hu[1, 2], Xiaohui Yang[1, 2], Lihong Hu[1, 2], Chengguo Liu[1, 2*], Xiaoli Ren[1, 2] and Yonghong Zhou[1, 2*]

[1] Institute of Chemical Industry of Forest Products, CAF; Key Lab. of Biomass Energy and Material; Key and Open Lab. of Forest Chemical Engineering, SFA; National Engineering Lab. for Biomass Chemical Utilization, No 16, Suojin Wucun, Nanjing, 210042, Jiangsu Province, China

[2] Co-Innovation Center of Efficient Processing and Utilization of Forest Resources, Nanjing Forestry University, 159 Longpan Road, Nanjing 210037, Jiangsu Province, China

[3] College of Biology and the Environment, Nanjing Forestry University, 159 Longpan Road, Nanjing 210037 Jiangsu Province, China

* Correspondence: liuchengguo@icifp.cn (C. Liu); zyh@icifp.cn (Y. Zhou); Tel.: (+86-025-85482520)

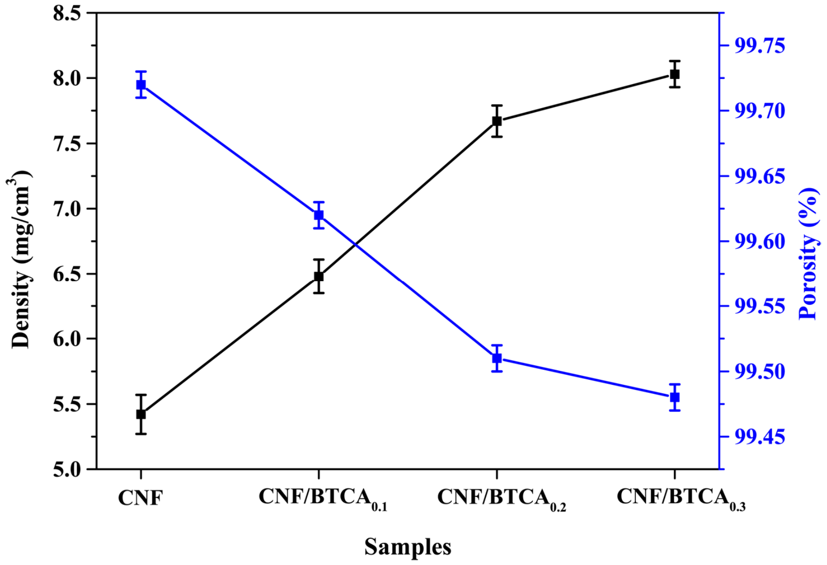

Figure S1 The variation of the density and porosity of the aeroges with the increasing BTCA dosage.
